# MiR-137 inhibited cell proliferation and migration of vascular smooth muscle cells via targeting IGFBP-5 and modulating the mTOR/STAT3 signaling

**DOI:** 10.1371/journal.pone.0186245

**Published:** 2017-10-10

**Authors:** Jin Pan, Kai Li, Wei Huang, Xiaoqing Zhang

**Affiliations:** 1 Clinical Medical College, Xi'an Medical University, Xi'an City, Shaanxi Province, China; 2 Department of Cardiology, the First Affiliated Hospital of Xi'an Medical University, Xi'an City, Shaanxi Province, China; Qatar University College of Health Sciences, QATAR

## Abstract

Abnormal proliferation of vascular smooth muscle cells (VSMCs) contributes to the development of cardiovascular diseases. Studies have shown the great impact of microRNAs (miRNAs) on the cell proliferation of VSMCs. This study examined the effects of miR-137 on the cell proliferation and migration of VSMCs and also explored the underlying molecular mechanisms. The mRNA and protein expression levels were determined by qRT-PCR and western blot assays, respectively. The CCK-8 assay, wound healing assay and transwell migration assay were performed to measure cell proliferation and migration of VSMCs. The miR-137-targeted 3’untranslated region of insulin-like growth factor-binding protein-5 (IGFBP-5) was confirmed by luciferase reporter assay. Platelet-derived growth factor-bb (PDGF-bb) treatment enhanced cell proliferation and suppressed the expression of miR-137 in VSMCs. The gain-of-function and loss-of-function assays showed that overexpression of miR-137 suppressed the cell proliferation and migration, and also inhibited the expression of matrix genes of VSMCs; down-regulation of miR-137 had the opposite effects on VSMCs. Bioinformatics analysis and luciferase report assay results showed that IGFBP-5 was a direct target of miR-137, and miR-137 overexpression suppressed the IGFBP-5 expression and down-regulation of miR-137 increased the IGFBP-5 expression in VSMCs. PDGF-bb treatment also increased the IGFBP-5 mRNA expression. In addition, enforced expression of IGFBP-5 reversed the inhibitory effects of miR-137 on cell proliferation and migration of VSMCs. More importantly, overexpression of miR-137 also suppressed the activity of mTOR/STAT3 signaling in VSMCs. Taken together, the results suggest that miR-137 may suppress cell proliferation and migration of VSMCs via targeting IGFBP-5 and modulating mTOR/STAT3 signaling pathway.

## Introduction

Cardiovascular diseases, including coronary artery disease, stroke, atherosclerosis, hypertension, myocardial infarction and stroke, are the leading cause of the death worldwide [1]. Mounting evidence has demonstrated that disruption of vascular smooth muscle cells (VSMCs) proliferation is a key factor in the development of cardiovascular diseases [2, 3]. Studies have shown that the abnormal elevation of growth regulating factors, cell factors and vasoactive substances under pathological conditions can promote the VSMCs proliferation and also alter the gene expression profiles of VSMCs [4]. Among these factors, platelet-derived growth factor (PDGF) is one of the most potent inducers for VSMCs proliferation and migration. PDGF-bb is primarily released by the vascular endothelial cells and platelets at the vascular injury sites, and PDGF-bb promotes VSMCs proliferation and migration via regulating the transcriptional factors and critical molecular signaling pathways [5–7]. However, the exact molecular mechanisms underlying VSMCs proliferation are largely unknown.

MicroRNAs (miRNAs) are a class of ∼22 nucleotide non-coding short RNAs and play important roles in cell proliferation, cell differentiation, metabolism and development [8, 9]. MiRNAs exert their functions by targeting the 3'untranslated region (3'UTR) of the targeted genes, which results in degradation of mRNA or repression of mRNA translation [10]. Aberrant miRNA expression has been linked to various diseases such as cancer and cardiovascular diseases, and studying the role of miRNAs in cardiovascular disease may be important for us to understand the molecular mechanisms underlying VSMCs proliferation. [11]. Previously, we have demonstrated that miR-379 was down-regulated after PDGF-bb treatment, and miR-379 was found to suppress the VSMCs proliferation, invasion and migration via targeting insulin-like factor-1 [12]. In addition, lots of miRNAs have been shown to have a regulatory role for VSMCs proliferation and migration. MiR-503 was found to inhibit PDGF-bb-induced human aortic VSMCs proliferation and migration via targeting the insulin receptor [13]. MiR-145 was found to have inhibitory effects on the VSMCs proliferation, and this inhibitory effect was mediated via targeting the CD40 [14]. On the other hand, miR-34a was found to promote proliferation of human pulmonary artery smooth muscle cells by targeting platelet-derived growth factor alpha [15], and miR-181b activated the PI3K and MAPK signaling pathways, which subsequently promoted VSMCs proliferation [16]. Recently, miR-137 was found to play tumor-suppressive roles in the different types of cancers [17–20]. However, it is unclear whether miR-137 plays a role in the VSMCs proliferation and migration.

In the present study, we showed that PDGF-bb suppressed the expression of miR-137 in VSMCs. *In vitro* functional studies found that miR-137 had inhibitory effects on the VSMCs proliferation and migration. Bioinformatics prediction and luciferase reporter assay showed that insulin-like growth factor-binding protein-5 (IGFBP-5) was a direct target of miR-137 in VSMCs. MiR-137 overexpression also suppressed the activity of mTOR/STAT3 signaling.

## Materials and methods

### Cell culture

VSMCs cell lines (human aortic smooth muscle cells, #6110) were from the ScienCell (San Diego, USA), and the cells were cultured in Dulbecco’s modified Eagle’s medium (DMEM; Hyclone, GE Health Care, USA) with 10% fetal bovine serum (FBS; Gibco, Thermo Fisher Scientific, Waltham, USA). Cells were cultured in a humidified atmosphere with 5% CO_2_ at 37°C.

### MiRNAs and plasmids

MiR-137 mimics, miR-137 inhibitor and their relative control miRNAs (mimics control and inhibitor control) were purchased from Ribobio (Guangzhou, China). The empty vector, pcDNA3.1, and the IGFBP-5 overexpressing vector, pcDNA3.1-IGFBP-5 were also purchased from Ribobio company.

### Transfection, platelet-derived growth factor-bb (PDGF-bb) treatment

The miRNAs and plasmids transfections were performed by using the Lipofectamine 2000 (Invitrogen, Carlsbad, USA) according to the manufacturer’s instructions. PDGF-bb (Sigma, St. Louis, USA) treatment was performed at a concentration of 30 ng/ml, 24 h, 48 h or 72 h after transfection, the VSMCs were subjected to CCK-8 assay or quantitative real-time PCR (qRT-PCR) assay.

### RNA preparation and qRT-PCR analysis

Total RNAs were isolated from VSMCs by using TRIzol reagent (Invitrogen). The PrimeScript RT Master Mix Kit (Takara, Dalian, China) was used to obtain cDNA for mRNA detection, whereas PrimeScript II 1st Strand cDNA Synthesis Kit (Takara) was applied to reverse transcribe RNA for miRNA detection. For miR-137 and other mRNAs, qRT-PCR was performed using SYBR Green PCR Kit (Takara) according to the manufacturer’s instructions, respectively. U6 was used as an internal control for miR-137, and GAPDH was used as an internal control for other genes including proteoglycan, type I collagen, type V collagen and IGFBP-5. Data were expressed as fold changes relative to U6 or GAPDH, and were calculated based on the following formula: RQ = 2^-ΔΔCt^.

### Cell proliferation assay

For the CCK-8 assay, cells were seeded in 96-well plates (5 x 10^3^ cells/well) and incubated for 24 h, and then treated with PDGF-bb; or transfected with miR-137 mimics, miR-137 inhibitor or their relative controls (mimics control or inhibitor control); or co-transfected with mimics control + pcDNA3.1, miR-137 mimics + pcDNA3.1, or miR-137 mimics + pcDNA3.1-IGFBP-5. At 0 h, 24 h, 48 h, and 72 h after PDGF-bb treatment, or at 48 h after transfection, CCK-8 kit (Beyotime, Beijing, China) was used to detect cell proliferation index according to manufacturer’s instructions.

### Wound healing assay

For the wound healing assay, cells were seeded in 6-well plates (5 x 10^5^ cells/well), 48 h after transfection, a pipette tip was used to create a wound. The cells were then cultured in serum-free medium. Cells migrated into wound surface and the average distance of migrating cells was determined under an inverted microscope at 0 h and 24 h. Area of wound healed (%) = (Wound area at 24 h—Wound are at 0 h)/Wound area at 0 h x 100%.

### Transwell migration assay

For the transwell migration assay, transfected cells were seeded on the upper transwell insert with 8 μm-pore membrane (Corning, Lowell, USA) containing serum-free medium, and the lower chamber was filled with DMEM medium supplemented with 10% FBS. After 24 h incubation, the cells on the upper surface of the insert were removed, and the cells on the lower surface of the insert were fixed with 4% paraformaldehyde and stained with 0.1% crystal violet. The number of migrated cells were counted under a light microscope (Zeiss, Oberkochen, Germany)

### Luciferase reporter assay

The pmirGLO vectors containing wild type or mutant miR-137 binding site in IGFBP-5 3’UTR were synthesized by Ribobio. VSMCs were seeded into 24-well plates 24 h before transfection and then co-transfected with 50 ng of wild type or mutant luciferase vector containing IGFBP-5 3’UTR and 20 μM miR-137 mimics, miR-137 inhibitor or their relative controls. After 48 h, luciferase activity was assayed by using the Dual-luciferase Reporter Assay System (Promega, Madison, USA).

### Western blot

Proteins were extracted from whole cell lysates and separated by sodium dodecyl sulfate-polyacrylamide gel electrophoresis, then transferred to a polyvinylidene fluoride membrane. The following primary antibodies were used: rabbit polyclonal anti-IGFBP-5 (1:1500; Abcam, Cambridge, UK), rabbit polyclonal anti-proteoglycan (1:1200, Abcam), rabbit polyclonal anti-type I collagen (1:1500, Abcam), rabbit polyclonal anti-type V collagen (1:2000, Abcam), rabbit monoclonal anti-phosphorylated mTOR (p-mOTR, 1:1000, Cell Signaling Technology, Danvers, USA), rabbit monoclonal anti-mTOR (1:1000, Cell Signaling Technology), rabbit monoclonal anti-phosphorylated STAT3 (p-STAT3, 1:2000, Cell signaling Technology), rabbit monoclonal anti-STAT3 (1:1000, Cell Signaling Technology), and rabbit monoclonal anti-β-actin (1: 5000; Cell Signaling Technology). Membranes were then incubated with the horseradish peroxidase-conjugated secondary antibodies (1:4000; Abcam). The ECL western blot substrate kit was used to detect the respective bands (Abcam). The membranes were exposed using a ChemoDoc XRS detection system (Bio-Rad, Milan, Italy).

### Statistical analysis

All statistical analysis was carried out using GraphPad Prism version 6 (La Jolla, USA). The differences among groups were analyzed by one-way ANOVA followed by Bonferroni’s multiple comparison tests or t-test. All data are expressed as the mean values of three independent replicates ± SD; differences were considered to be statistically significant when P<0.05.

## Results

### PDGF-bb promoted cell proliferation and down-regulated the expression of miR-137 in VSMCs

**Fig 1:**
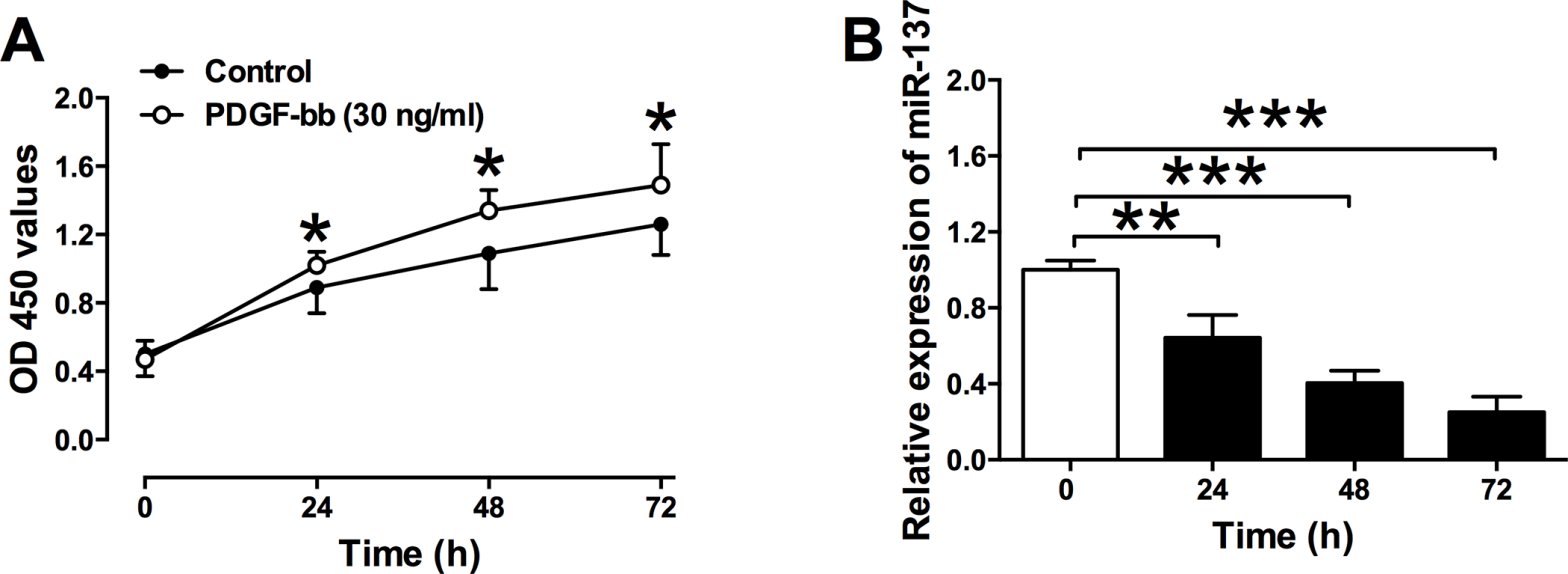
Effects of PDGF-bb on cell proliferation and miR-137 expression in VSMCs. (A) Cell proliferation of VSMCs was determined by the CCK-8 assay, and PDGF-bb (30 ng/ml) treatment enhances cell proliferation of VSMCs. (B) The relative expression of miR-137 was determined by qRT-PCR, and PDGF-bb (30 ng/ml) treatment suppresses the miR-137 expression in a time-dependent manner. Data represents the mean values of three independent replicates; significant differences between groups were shown as *P<0.05, **P<0.01, ***P<0.001.

The effects of PDGF-bb on the cell proliferation of VSMCs were examined by CCK-8 assay. PDGF-bb (30 ng/ml) treatment significantly increased the cell proliferation of VSMCs when compared to the control group (Fig 1A). The effects of PDGF-bb on the expression of miR-137 in VSMCs were also examined by qRT-PCR, and PDGF-bb (30 ng/ml) significantly suppressed the relative expression level of miR-137 and this effect was in a time-dependent manner (Fig 1B).

### MiR-137 inhibited cell proliferation and migration of VSMCs

**Fig 2:**
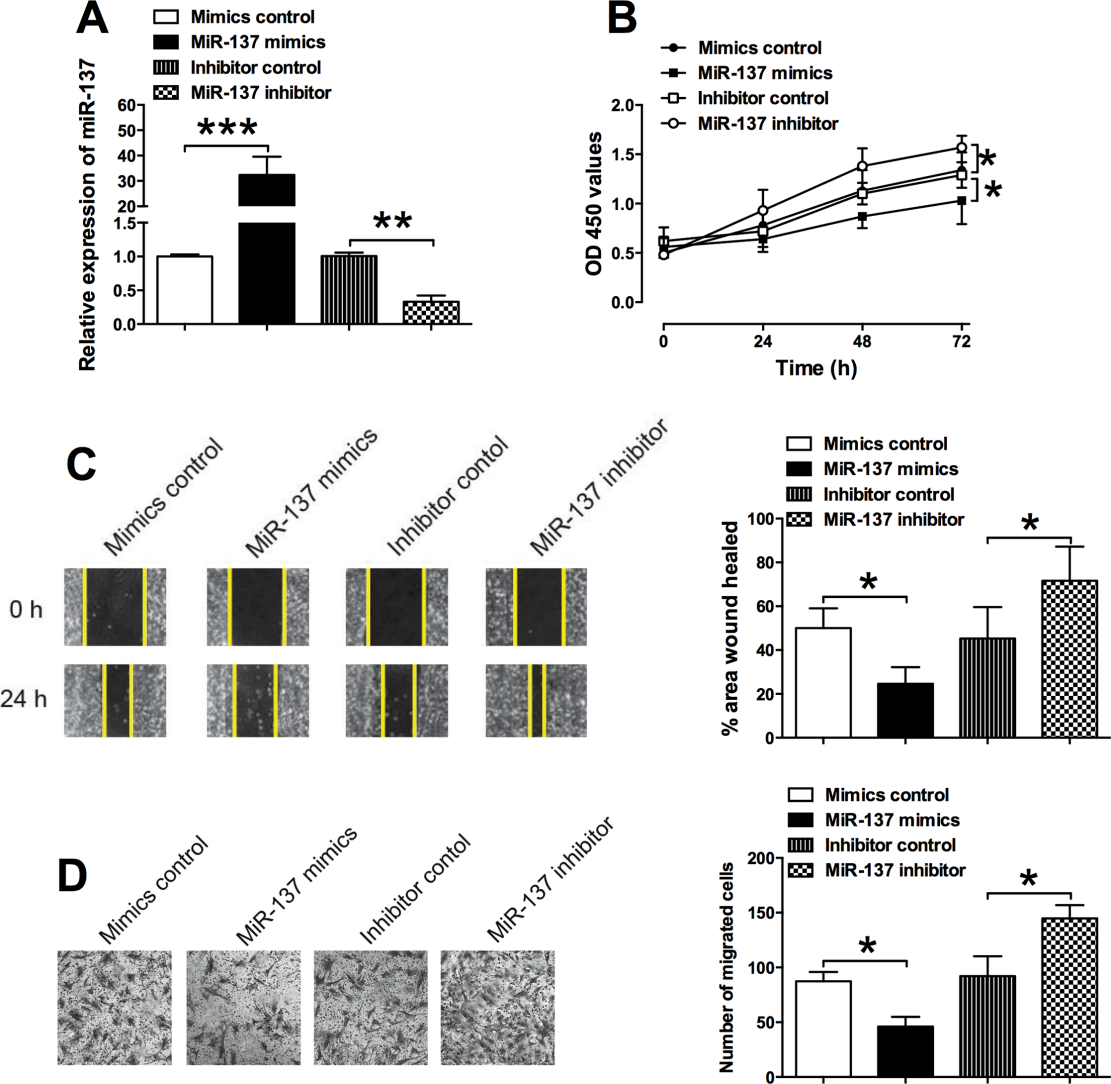
Effects of miR-137 on cell proliferation and cell migration of VSMCs. (A) The relative expression of miR-137 in VSMCs after miR-137 mimics, miR-137 inhibitor or their relative controls transfection was determined by qRT-PCR. (B) The cell proliferation of VSMCs after miR-137 mimics, miR-137 inhibitor or their relative controls transfection was assessed by the CCK-8 assay. The cell migration of VSMCs after miR-137 mimics, miR-137 inhibitor or their relative controls transfection was assessed by (C) the wound healing assay and (D) transwell migration assay. Data represents the mean values of three independent replicates; significant differences between groups were shown as *P<0.05, **P<0.01, ***P<0.001.

**Fig 3:**
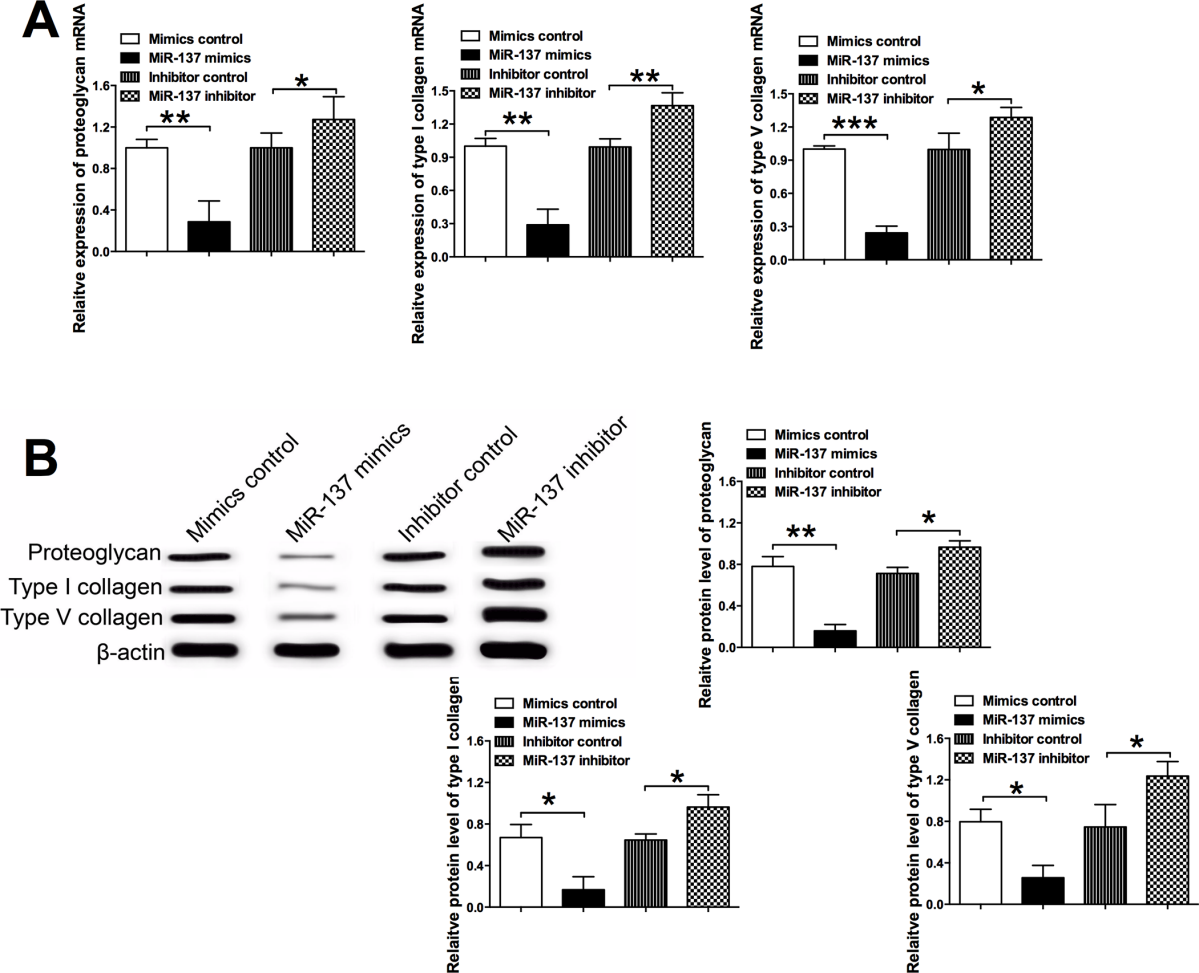
Effects of miR-137 on the matrix gene expression in VSMCs. (A) The relative mRNA expression levels of proteoglycan, type I collagen and type V collagen in VSMCs after miR-137 mimics, miR-137 inhibitor or their relative controls transfection were determined by qRT-PCR. (B) The relative protein levels of proteoglycan, type I collagen and type V collagen in VSMCs after miR-137 mimics, miR-137 inhibitor or their relative controls transfection were examined by the western blot assay. Data represents the mean values of three independent replicates; significant differences between groups were shown as *P<0.05, **P<0.01, ***P<0.001.

The *in vitro* function of miR-137 in VSMCs was also explored by the CCK-8 assay, wound healing assay and transwell migration assay. Firstly, the VSMCs were transiently transfected with miR-137 mimics, miR-137 inhibitor or their relative controls (mimics control or inhibitor control), and miR-137 mimics transfection significantly increased the miR-137 expression by more than 30 fold when compared to mimics control group; while transfection with miR-137 inhibitor significantly suppressed the miR-137 expression level when compared to the inhibitor control group (Fig 2A). CCK-8 assay showed that compared to the mimics control group, the cell proliferation of VSMCs was significantly suppressed in the miR-137 mimics transfection group; and miR-137 inhibitor transfection further promoted the cell proliferation of VSMCs when compared to inhibitor control group (Fig 2B), suggesting the inhibitory effect of miR-137 on the cell proliferation of VSMCs. The cell migration of VSMCs after miRNAs transfection was examined by the wound healing assay and transwell migration assay, as shown in Fig 3C, miR-137 mimics transfection significantly reduced the wound closure area when compared to VSMCs transfected with mimics control (Fig 2C); while miR-137 inhibitor transfection significantly accelerated the wound closure of VSMCs when compared to inhibitor control group (Fig 2C). Consistently, the transwell migration assay showed that overexpression of miR-137 decreased the number of migrated cells of VSMCs (Fig 2D), and knock-down of miR-137 increased the number of migrated cells of VSMCs (Fig 2D). These results may imply the inhibitory effects of miR-137 on the cell migration of VSMCs.

### MiR-137 suppressed the expression of matrix genes in VSMCs

The effects of miR-137 on the mRNA and protein levels of matrix genes, including proteoglycan, type I collagen, type V collagen, were determined by qRT-PCR and western blot assays. For the qRT-PCR assay, miR-137 mimics transfection significantly suppressed the mRNA expression levels of proteoglycan, type I collagen and type V collagen in VSMCs when compared to the mimics control transfection, and miR-137 inhibitor transfection had the opposite effects, where the mRNA expression levels of proteoglycan, type I collagen and type V collagen were significantly higher in the miR-137 inhibitor group than that in inhibitor control group (Fig 3A). Consistently, western blot assay showed overexpression of miR-137 by miR-137 mimics transfection suppressed the protein levels of proteoglycan, type I collagen, and type V collagen; while down-regulation of miR-137 by miR-137 inhibitor control transfection significantly increased protein levels of proteoglycan, type I collagen and type V collagen (Fig 3B). These results suggest that miR-137 had inhibitory effects on the expression of matrix genes in VSMCs.

### MiR-137 targets the 3’TUR of IGFBP-5

**Fig 4:**
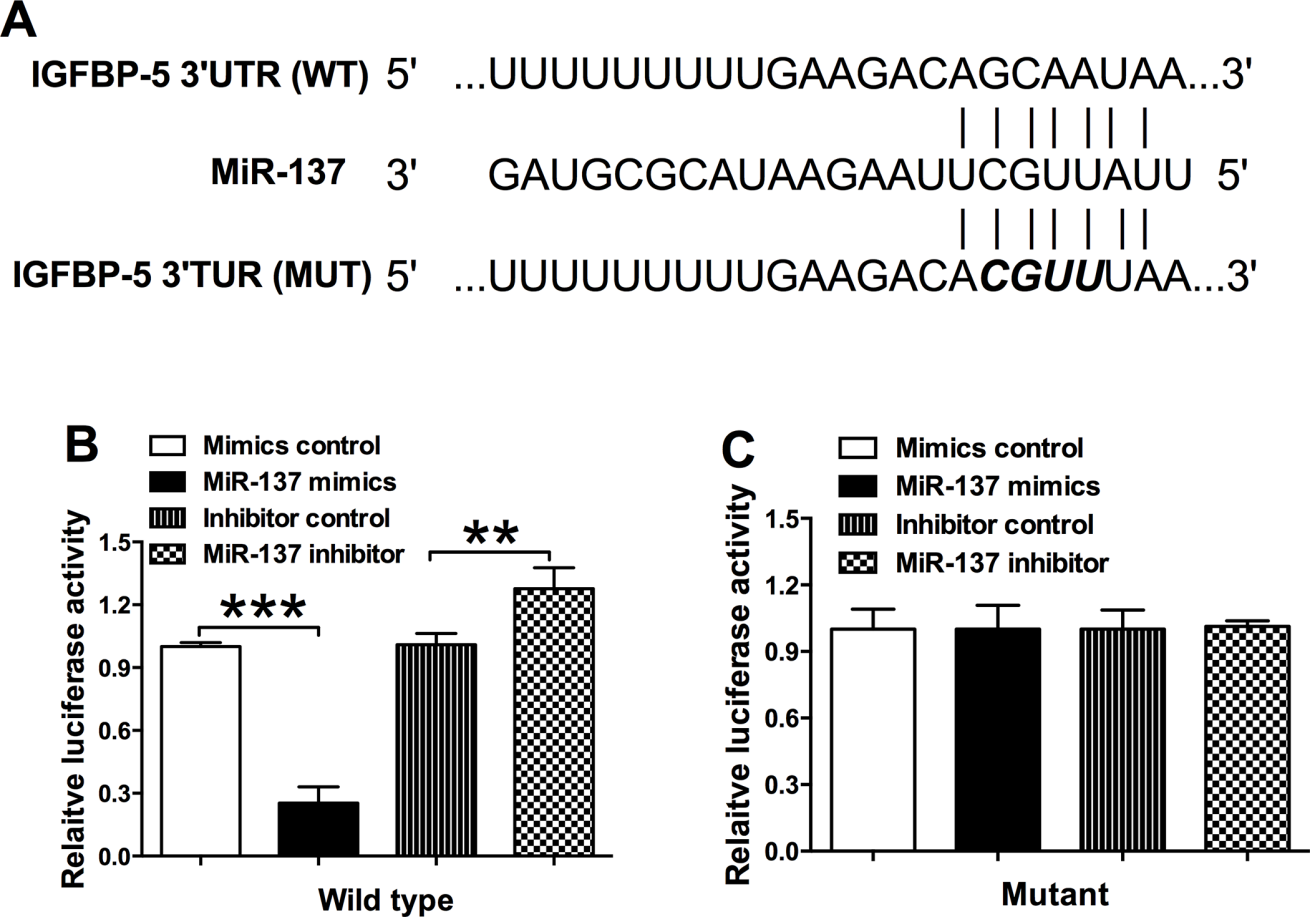
MiR-137 targets the 3'UTR of IGFBP-5 in VSMCs. (A) The seed sequence of wild type (WT) 3’UTR or mutant (Mut) 3’UTR of IGFBP-5 that is targeted miR-137; (B) and (C) VSMCs were co-transfected with miR-137 mimics, miR-137 inhibitor or their relative controls with wild type or mutant 3’UTR of IGFBP-5, and luciferase activity was detected. Data represents the mean values of three independent replicates; significant differences between groups were shown as **P<0.01, ***P<0.001.

The downstream targets of miR-137 were predicted by bioinformatics analysis, and among the predicted targets of miR-137, IGFBP-5 was chosen for further exploration. To validate whether IGFBP-5 is a target of miR-137, the IGFBP-5 3’UTR fragment containing wild type or mutant miR-137-binding sequence was sub-cloned to the Firefly luciferase reporter gene (Fig 4A). When miR-137 mimics were co-transfected with the wild type reporter plasmid, the relative luciferase activity of the reporter containing wild type IGFBP-5 3’ UTR was significantly suppressed in VSMCs; and co-transfection of miR-137 inhibitor and the wild type report plasmid significantly increased the luciferase activity of the reporter containing wild type IGFBP-5 3’ UTR in VSMCs (Fig 4B); while the luciferase activity of the reporter containing mutant 3’UTR of IGFBP-5 after co-transfection with different miRNAs and mutant reporter plasmids was unaffected (Fig 4C).

### MiR-137 suppressed the expression of IGFBP-5 in VSMCs

**Fig 5:**
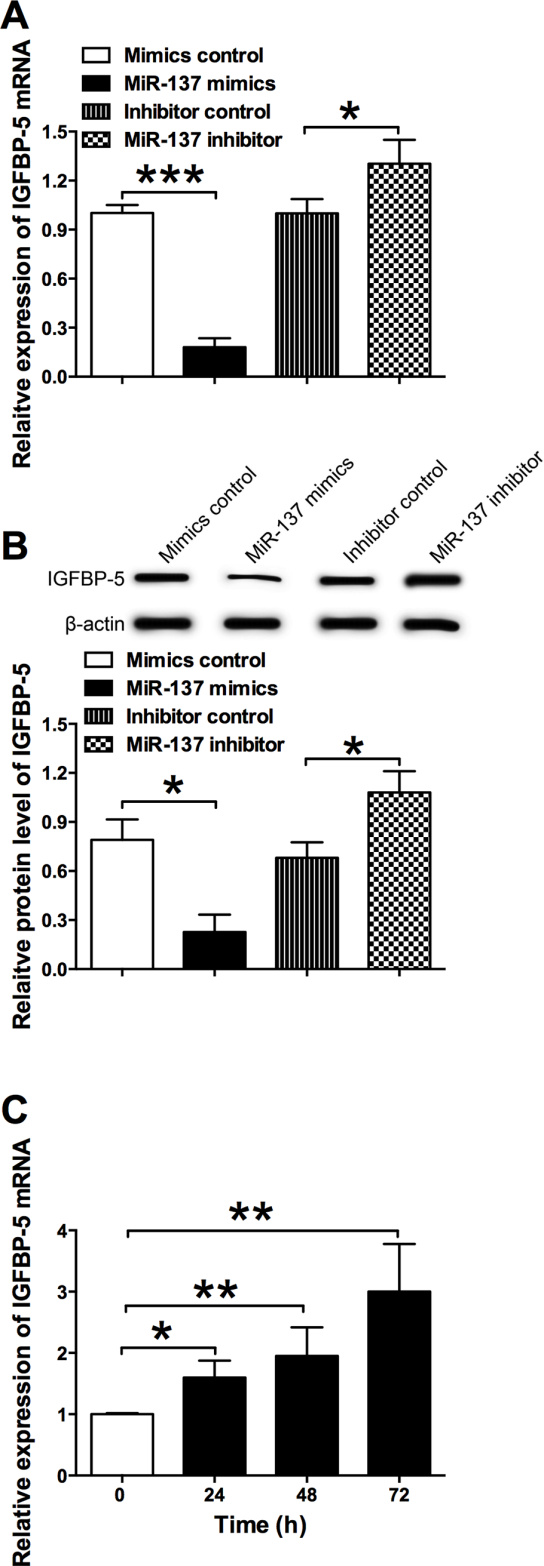
Effects of miR-137 on the IGFBP-5 expression in VSMCs. (A) The relative mRNA expression levels of IGFBP-5 in VSMCs after miR-137 mimics, miR-137 inhibitor or their relative controls transfection were determined by qRT-PCR. (B) The relative protein expression levels of IGFBP-5 in VSMCs after miR-137 mimics, miR-137 inhibitor or their relative controls transfection were determined by the western blot assay. (C) The relative mRNA expression levels of IGFBP-5 in VSMCs after PDGF-bb (30 ng/ml) treatment were determined by qRT-PCR. Data represents the mean values of three independent replicates; significant differences between groups were shown as *P<0.05, ***P<0.001.

The effects of miR-137 on the mRNA and protein expression of IGFBP-5 were examined by qRT-PCR and western blot assays. As shown in Fig 5A, overexpression of miR-137 by miR-137 mimics transfection significantly suppressed the mRNA expression level of IGFBP-5 when compared to mimics control transfection in VSMCs; and down-regulation of miR-137 by miR-137 inhibitor transfection increased the mRNA expression level of IGFBP-5 when compared to inhibitor control transfection in VSMCs (Fig 5A). In agreement with the qRT-PCR results, the western blot assay showed that the protein level of IGFBP-5 in the miR-137 mimics transfection was significantly lower in VSMCs than that from mimics control group, and the protein level of IGFBP-5 in miR-137 inhibitor group was significantly increased when compared to the inhibitor control group (Fig 5B). PDGF-bb (30 ng/ml) also significantly increased the IGFBP-5 mRNA expression levels in VSMCs (Fig 5C). The results suggest that miR-137 has an inhibitory effect on the expression of IGFBP-5.

### IGFBP-5 reversed the inhibitory effects of miR-137 overexpression on cell proliferation and migration in VSMCs

**Fig 6:**
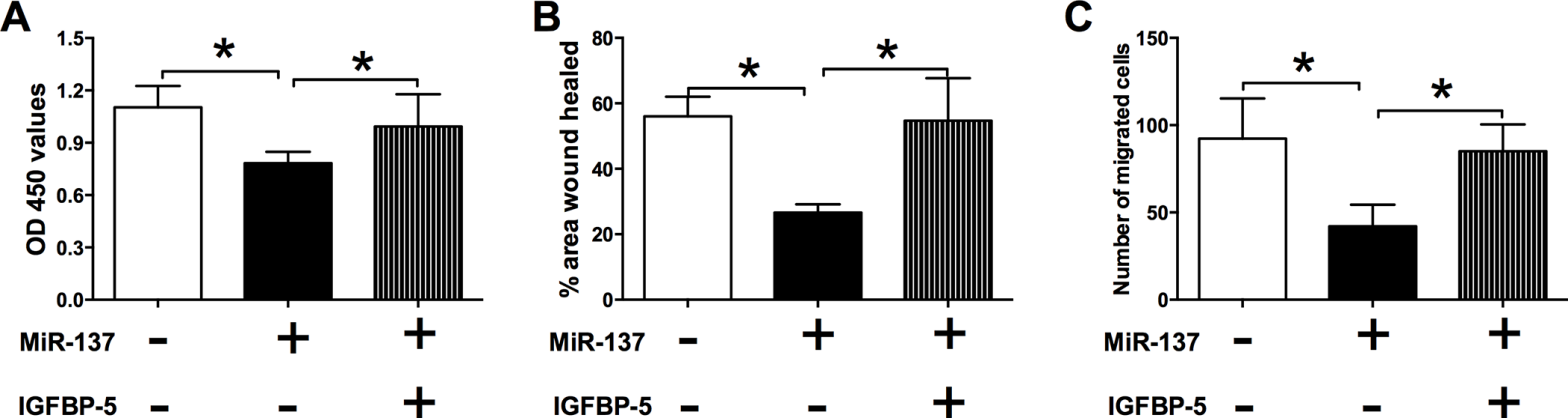
Effects of IGFBP-5 on the miR-137 mimics-induced changes in cell proliferation and cell migration of VSMCs. (A) The cell proliferation of VSMCs after co-transfection with mimics control + pcDNA3.1, miR-137 mimics + pcDNA3.1, or miR-137 mimics + pcDNA3.1-IGFBP-5 was determined by the CCK-8 assay. The cell migration of VSMCs after co-transfection with mimics control + pcDNA3.1, miR-137 mimics + pcDNA3.1, or miR-137 mimics + pcDNA3.1-IGFBP-5 was determined by (B) the wound healing assay and (C) transwell migration assay. Data represents the mean values of three independent replicates; significant differences between groups were shown as *P<0.01.

In order to further confirm the interaction between miR-137 and IGFBP-5, the VSMCs were co-transfected with miR-137 mimics and the IGFBP-5-overexpressing vector, pcDNA3.1-IGFBP-5, or their relative controls. For the CCK-8 assay, co-transfection with miR-137 mimics and pcDNA3.1 plasmid significantly suppressed the cell proliferation of VSMCs, and enforced expression of IGFBP-5 by pcDNA3.1-IGFBP-5 transfection reversed the inhibitory effect of miR-137 overexpression on the cell proliferation (Fig 6A). For the wound healing assay, the area of healed wound was significantly larger in VSMCs co-transfected with miR-137 mimics and pcDNA3.1-IGFBP-5 than that co-transfected with miR-137 mimics and pcDNA3.1 (Fig 6B). Consistently, for the transwell migration assay, the number of migrated cells was significantly higher in VSMCs co-transfected with miR-137 mimics and pcDNA3.1-IGFBP-5 than that co-transfected with miR-137 mimics and pcDNA3.1 (Fig 6C). The results suggest that IGFBP-5 reversed the inhibitory effects of miR-137 overexpression on cell proliferation and migration of VSMCs.

### MiR-137 suppressed the mTOR/STAT3 signaling in VSMCs

**Fig 7:**
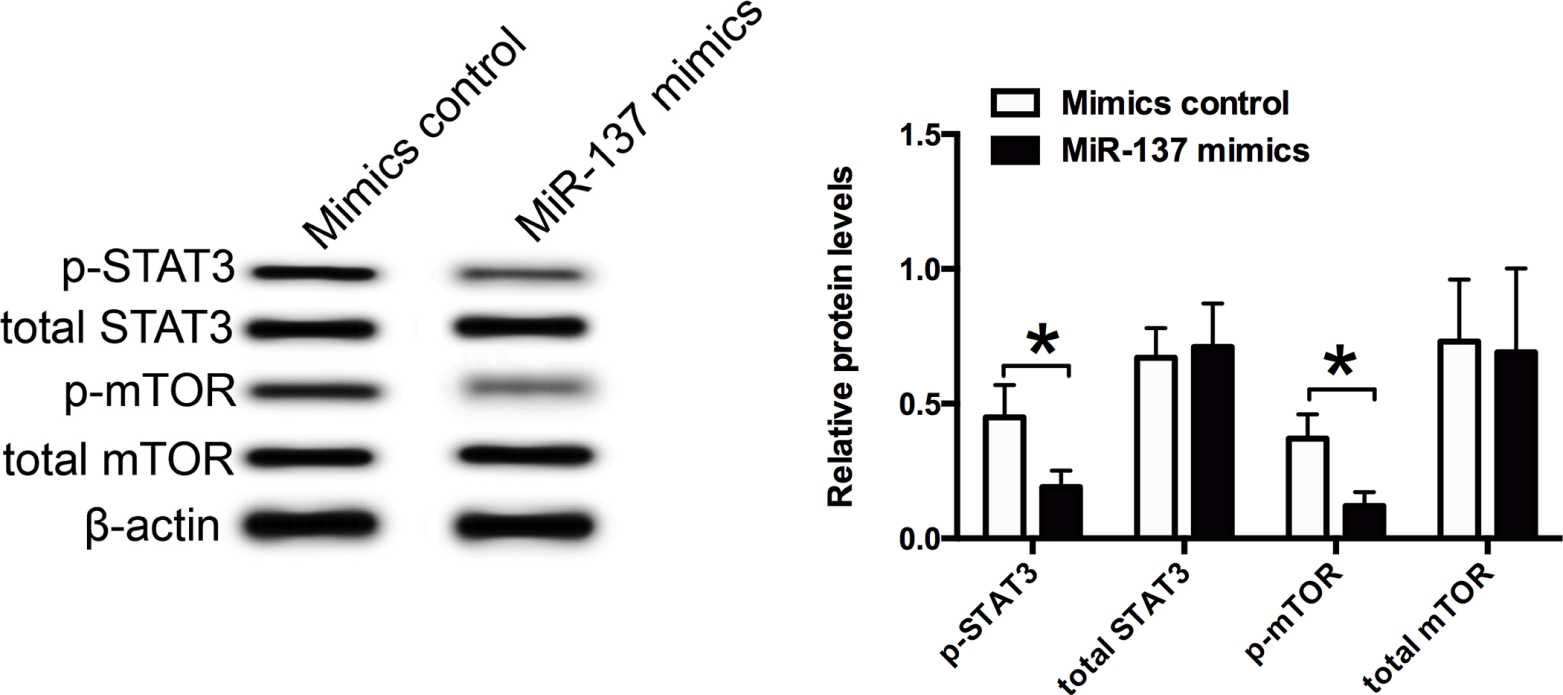
Effects of miR-137 on the mTOR/STAT3 signaling pathway in VSMCs. The relative protein expression levels of p-STAT3, total STAT3, p-mTOR and total mTOR in VSMCs after miR-137 mimics, miR-137 inhibitor or their relative controls transfection were determined by the western blot assay. Data represents the mean values of three independent replicates; significant differences between groups were shown as *P<0.05.

The effects of miR-137 on the mTOR/STAT3 signaling in VSMCs were examined by the western blot assay, and the protein levels of p-STAT3, total STAT3, p-mOTR and total mTOR were assessed. As shown in Fig 7, miR-137 mimics transfection significantly suppressed the protein levels of p-STAT3 and p-mTOR when compared to the mimics control group, and miR-137 mimics transfection had no effect on the protein levels of total STAT3 and total mTOR, suggesting the miR-137 suppresses the activity of mTOR/STAT3 signaling in VSMCs.

## Discussion

Aberrant proliferation of VSMCs has been shown to contribute to the development of vascular diseases, and understanding of molecular mechanisms underlying the abnormal proliferation of VSMCs may be essential for us to identify new therapeutic targets for the management of cardiovascular diseases. In this study, our results showed that PDGF-bb treatment enhanced the cell proliferation and suppressed the expression of miR-137 in VSMCs. The gain-of-function and loss-of-function assays showed that overexpression of miR-137 suppressed the cell proliferation and migration, and also inhibited the expression of matrix genes of VSMCs; down-regulation of miR-137 enhanced cell proliferation and migration, and increased the expression of matrix genes of VSMCs. Bioinformatics analysis and luciferase report results showed that IGFBP-5 was a direct target of miR-137, and miR-137 overexpression suppressed the IGFBP-5 expression and down-regulation of miR-137 increased IGFBP-5 expression in VSMCs. In addition, enforced expression of IGFBP-5 reversed the inhibitory effects of miR-137 on cell proliferation and migration of VSMCs. More importantly, overexpression of miR-137 also suppressed the activity of mTOR/STAT3 signaling in VSMCs. These results suggest that miR-137 suppressed cell proliferation and migration of VSMCs via targeting IGFBP-5 and modulating mTOR/STAT3 signaling pathway.

PDGF-bb is primarily released by the vascular endothelial cells and platelets at the vascular injury sites, and PDGF-bb promotes VSMCs proliferation and migration via regulating the transcriptional factors and critical molecular signaling pathways [5, 21]. Recently, studies have demonstrated that PDGF-bb treatment differentially regulated the expression of miRNAs in VSMCs. In some studies, PDGF-bb treatment induced the up-regulation of miRNAs such as miR-15b, miR-541 and miR-146b-5p [22–24]; while in other experiments, PDGF-bb treatment suppressed the expression of miRNAs such as miR-599, miR-21 and miR-145 [14, 25, 26]. In the present study, we showed that PDGF-bb treatment suppressed the expression of miR-137. The role of miR-137 has been demonstrated in various cancer studies. MiR-137 promoted apoptosis in ovarian cancer cells via targeting the X-linked inhibitor of apoptosis protein [19]. MiR-137 was also found to play a tumor-suppressive role in gastric cancer cell lines via interacting with Kruppel like factor 12 and myosin IC [27]. MiR-137 inhibited proliferation and angiogenesis of human glioblastoma cells by targeting enhancer of zeste homolog 2 [28]. These results suggest that miR-137 may have a suppressive role in the cell proliferation of cancer cells. Similarly, our data showed that overexpression of miR-137 inhibited cell proliferation and migration, and the expression of matrix genes of VSMCs, and down-regulation of miR-137 enhanced the cell proliferation and migration, and the expression of matrix genes of VSMCs, suggesting that miR-137 had an inhibitory effect on the cell proliferation and migration of VSMCs.

IGFBP-5 belongs to the IGF-BPs family, which binds IGFs and modulates IGF distribution, stability and biological activities [29–31]. Studies have shown that VSMCs can synthesize and secrete IGFBP-5, and IGFBP-5 was found to be a nuclear protein and promoted cell proliferation and migration of VSMCs independent of IGF [31, 32]. In the present study, bioinformatics prediction and luciferase reporter assay showed that miR-137 can target the 3’TUR of IGFBP-5, and overexpression of miR-137 suppressed the expression of IGFBP-5, while down-regulation of miR-137 increased the expression of IGFBP-5 in VSMCs. Consistently, the enforced expression of IGFBP-5 reversed the inhibitory effects of miR-137 on the cell proliferation and migration of VSMCs. These results suggested that miR-137 suppressed the cell proliferation and migration of VSMCs via down-regulation of IGFBP-5.

The role of mTOR/STAT3 signaling in cell proliferation and migration has been implicated in various studies [33, 34]. Activation of mTOR/STAT3 signaling has been linked to the development and progression of cancers such as breast cancer, gastric cancer and liver cancer [33, 35, 36]. Li et al., showed that activated monocytes stimulated the cell proliferation of VSMCs via activating the mTOR/STAT3 signaling pathway [37]. In the cancer studies, miR-137 was found to exert its anti-tumor activity via inhibiting the AKT2/mTOR signaling pathway in the liver cancer [38]. In the gastric cancer, miR-137 functioned as a tumor suppressor via targeting Cox-2-activiated PI3K/AKT signaling pathway [39]. In the present study, we found that miR-137 overexpression suppressed the expression of p-STAT3 and p-mTOR, suggesting that miR-137 suppresses the cell proliferation and migration of VSMCs via targeting the mTOR/STAT3 signaling.

Taken together, our results establish a functional link between miR-137 and IGFBP-5 expression in VSMCs, demonstrating that IGFBP-5 is directly suppressed by miR-137, which suppressed the cell proliferation and migration. The present study advances our knowledge into understanding the molecular mechanisms underlying the miR-137-regulated VSMCs proliferation.
